# Regular Use of VKA Prior to COVID-19 Associated with Lower 7-Day Survival in Hospitalized Frail Elderly COVID-19 Patients: The GERIA-COVID Cohort Study

**DOI:** 10.3390/nu13010039

**Published:** 2020-12-24

**Authors:** Pierre Ménager, Olivier Brière, Jennifer Gautier, Jérémie Riou, Guillaume Sacco, Antoine Brangier, Cédric Annweiler, on behalf of the GERIA-COVID study group

**Affiliations:** 1 Department of Geriatric Medicine, Le Mans Hospital, F-72037 Le Mans, France; pierrebenoitmenager@yahoo.fr; 2 Department of Geriatric Medicine and Memory Clinic, Research Center on Autonomy and Longevity, University Hospital, F-49933 Angers, France; olivierbriere53@gmail.com (O.B.); jegautier@chu-angers.fr (J.G.); yogisacco@gmail.com (G.S.); Antoine.Brangier@chu-angers.fr (A.B.); 3 INSERM, MINT, 1066, University of Angers, F-49000 Angers, France; jeremie.riou@univ-angers.fr; 4 Delegation to Clinical Research and Innovation, Angers University Hospital, F-49933 Angers, France; 5 UPRES EA 4638, University of Angers, F-49000 Angers, France; 6 Gérontopôle Autonomie Longévité des Pays de la Loire, F-44000 Nantes, France; 7 Robarts Research Institute, Department of Medical Biophysics, Schulich School of Medicine and Dentistry, The University of Western Ontario, London, ON N6A 5K8, Canada

**Keywords:** COVID-19, SARS-CoV-2, vitamin K antagonist, anticoagulation, survival, older adults

## Abstract

Background. Vitamin K concentrations are inversely associated with the clinical severity of COVID-19. The objective of this cohort study was to determine whether the regular use of vitamin K antagonist (VKA) prior to COVID-19 was associated with short-term mortality in frail older adults hospitalized for COVID-19. Methods. Eighty-two patients consecutively hospitalized for COVID-19 in a geriatric acute care unit were included. The association of the regular use of VKA prior to COVID-19 with survival after 7 days of COVID-19 was examined using a propensity-score-weighted Cox proportional-hazards model accounting for age, sex, severe undernutrition, diabetes mellitus, hypertension, prior myocardial infarction, congestive heart failure, prior stroke and/or transient ischemic attack, CHA2DS2-VASc score, HAS-BLED score, and eGFR. Results. Among 82 patients (mean ± SD age 88.8 ± 4.5 years; 48% women), 73 survived COVID-19 at day 7 while 9 died. There was no between-group difference at baseline, despite a trend for more frequent use of VKA in those who did not survive on day 7 (33.3% versus 8.2%, *p* = 0.056). While considering “using no VKA” as the reference (hazard ratio (HR) = 1), the HR for 7-day mortality in those regularly using VKA was 5.68 [95% CI: 1.17; 27.53]. Consistently, COVID-19 patients using VKA on a regular basis had shorter survival times than the others (*p* = 0.031). Conclusions. Regular use of VKA was associated with increased mortality at day 7 in hospitalized frail elderly patients with COVID-19.

## 1. Introduction

The coronavirus disease 2019 (COVID-19) caused by the SARS-CoV-2 has been spreading worldwide since December 2019, affecting millions of people of all ages. While the majority of patients exhibit only mild symptoms [1], older adults have a worse prognosis, either due to the onset of acute respiratory distress syndrome (ARDS) [2] or due to other manifestations, including coagulopathy and thromboembolic disease [2,3].

Interestingly, previous studies have suggested that vitamin K concentrations are decreased in patients with COVID-19 compared to uninfected controls and are inversely associated with the clinical severity of COVID-19 [4]. Such poor COVID-19 outcomes accompanying low vitamin K concentrations may be explained by the deregulation of coagulation in the case of low vitamin K status, the increase in circulating levels of inflammatory cytokines, the onset of lung fibrosis, and by the promotion of comorbidities such as hypertension or diabetes mellitus that worsen the prognosis of COVID-19 [1,5,6].

In this perspective, it is noticeable that the regular use of vitamin K antagonists (VKAs) dramatically decreases the bioavailability of active vitamin K. VKAs are drugs commonly used for the prophylaxis and treatment of thromboembolic disease in older adults [7,8]. However, data on COVID-19 outcomes within the patients using VKA on a regular basis are still lacking. While VKAs are useful—but not the only drug option—to prevent thromboembolic complications in COVID-19 [9], we are concerned about the reduced vitamin K concentrations they cause and the possible life-threatening implications during COVID-19. We hypothesized that, by decreasing the bioavailability of active vitamin K, VKAs might be accompanied by reduced survival in older patients with COVID-19. The objective of the present cohort study was to determine whether the regular use of VKA prior to COVID-19 was associated with increased mortality compared to not using VKA among frail older adults hospitalized for COVID-19.

## 2. Materials and Methods

### 2.1. Study Population

The GERIA-COVID study consisted of a longitudinal observational study in one French geriatric acute care unit dedicated to COVID-19 patients during the first wave (ClinicalTrials.gov Identifier: NCT04560608). Data of the GERIA-COVID study were retrospectively collected from hospital records. The inclusion criteria in the study were as follows: (1) patients hospitalized in the geriatric acute care unit of the University Hospital of Angers, France, in March–June 2020; (2) no objection from the patient and/or relatives to the use of anonymized clinical and biological data for research purpose. The inclusion criteria for the present analysis were as follows: (1) diagnosis of COVID-19 with RT–PCR and/or chest CT-scan; (2) age 80 and over; (3) data available on the regular use of VKA and on the heart rhythm on hospital admission; (4) no introduction of VKA during the hospitalization for COVID-19; (5) data available on the vital status within 7 days after the diagnosis of COVID-19. Ninety-seven patients were consecutively diagnosed with COVID-19 during the study period in the geriatric acute care unit and were recruited in the GERIA-COVID study. Among them, 5 were aged less than 80 years, 1 had VKA started during the hospitalization, 9 had missing data on heart rhythm. Finally, 82 patients could be included in the present analysis.

### 2.2. Regular Use of Vitamin K Antagonists

The regular use of VKA (i.e., warfarin or acenocoumarol or fluindione) was noted from family physician prescriptions and sought by questioning the patients and relatives, regardless of the prescription period, the reason for anticoagulation and the history of the international normalized ratio (INR).

### 2.3. Outcome: 7-Day Mortality in COVID-19 Patients

The main outcome was the 7-day all-cause mortality. Follow-up started from the day of COVID-19 diagnosis for each patient and continued for 7 days or until death when applicable. This follow-up period was covered by the hospitalization for all patients.

### 2.4. Covariables

Covariables were age, sex, functional abilities, history of cancer, severe undernutrition, diabetes mellitus, hypertension, cardiomyopathy, myocardial infarction, non-sinus heart rhythm, congestive heart failure, stroke and/or transient ischemic attack, CHA2DS2-VASc score for atrial fibrillation stroke risk, HAS-BLED score for major bleeding risk, use of antibiotics and/or of pharmacological treatments of respiratory disorders, and estimated glomerular filtration rate (eGFR).

Functional abilities prior to COVID-19 were measured from 1 to 6 (best) with the iso-resources groups (GIR) [10]. History of hematological and solid cancers, diabetes mellitus, of hypertension, of cardiomyopathy, of myocardial infarction, of congestive heart failure, and of stroke and/or transient ischemic attack were noted from the medical register and by interviewing patients, their relatives and family physicians. Severe undernutrition was defined as serum albumin concentration < 30 g/L at the time of COVID-19 diagnosis. The heart rhythm was analyzed from the electrocardiogram on hospital admission. All non-sinus rhythms were identified, including atrial fibrillation. The risks of stroke and of major bleeding in atrial fibrillation were estimated using the consensual CHA2DS2-VASc and HAS-BLED scores, respectively [11]. The use of antibiotics (i.e., quinolones, beta-lactams, sulfonamides, macrolides, lincosamides, aminoglycosides, among others) and/or pharmacological treatments of respiratory disorders (i.e., beta2-adrenergic agonists, inhaled corticosteroids, antihistamines, among others) were noted from prescriptions during hospitalization. eGFR was estimated with the modification of diet in renal disease (MDRD) study equation using the measure of serum creatinine concentration collected at the time of the diagnosis of COVID-19.

### 2.5. Statistical Analysis

The participants’ characteristics were summarized using medians and interquartile ranges (IQR) or numbers and percentages, as appropriate. Comparisons between participants separated according to the vital status at day 7 of the diagnosis of COVID-19 were performed using the nonparametric Mann–Whitney test or Fisher’s exact test, as appropriate.

Weighting-based propensity scores (inverse probability weighting) were used to balance covariables between the participants regularly using VKA and the others. All major variables were taken into account: age, sex, severe undernutrition, diabetes mellitus, hypertension, prior myocardial infarction, congestive heart failure, prior stroke and/or transient ischemic attack, CHA2DS2-VASc score, HAS-BLED score, and eGFR. The balance between groups was performed using the average treatment effect (ATE) [12]. Once the profiles of participants were balanced between the two groups, a propensity-score-weighted Cox proportional-hazards model was performed to determine the survival during the 7-day follow-up according to the use of VKA prior to COVID-19.

A sensitivity analysis was also conducted in order to confirm the results obtained with the propensity score, using a more classical approach based on multiple Cox models. Unadjusted, partially adjusted (accounting for age, sex, functional ability and non-sinus heart rhythm) and fully adjusted (accounting for age, sex, functional abilities, history of cancer, hypertension, cardiomyopathy, non-sinus heart rhythm, use of antibiotics and/or of pharmacological treatments of respiratory disorders, and eGFR) Cox regressions were used to examine the associations of the regular use of VKA (independent variable) with the 7-day mortality (dependent variable). Scaled Schoenfeld residuals were computed to check the proportionality assumption. A statistical test was performed to validate the non-proportionality combined with a plot of these residuals against time. Hazard ratios (HR) with 95% confidence intervals (95% CI) were reported.

Finally, the relapsed time to death was illustrated by survival curves computed without propensity score adjustment, according to Kaplan–Meier estimator.

*p*-Values < 0.05 were considered significant. All statistics were performed using SAS^®^ version 9.4 software (SAS Institute Inc.) and R (R core team, 2018).

### 2.6. Ethics

The study was conducted in accordance with the ethical standards set forth in the Helsinki Declaration (1983). All participants and relatives were informed in writing and orally of the data collection, and none objected to the use of anonymized clinical and biological data for research purposes, as approved by the Ethics Board of the University Hospital of Angers, France (2020/100). The study protocol was also declared to the National Commission for Information Technology and Civil Liberties (CNIL; ar20-0087v0).

## 3. Results

Eighty-two participants (mean ± SD age 88.8 ± 4.5 years; 48% women; 11% using VKA prior to COVID-19; none using direct oral anticoagulant (DOAC); 24.4% using an antiplatelet agent; none using both VKA and antiplatelet agent) were included in the present analysis. A switch to heparin anticoagulant therapy (either low molecular weight heparin or unfractionated heparin depending on renal function) was made for all participants using VKA upon diagnosis of COVID-19, as recommended [13]. Finally, 73 participants survived COVID-19 on day 7, while 9 died.

Table 1 indicates the characteristics of participants separated according to survival at day 7. There was no between-group difference, despite a trend for more frequent use of VKA prior to COVID-19 in those who did not survive on day 7 (33.3% versus 8.2%, *p* = 0.056).

After appropriately adjusting for the propensity score, the weighted Cox model found a direct association between the regular use of VKA prior to COVID-19 and the 7-day mortality. While considering “using no VKA” as the reference (HR = 1), the HR for mortality in those using VKA on a regular basis was 5.68 [95% CI: 1.17; 27.53] (*p* = 0.0312).

The sensitivity analysis using the unadjusted, partially adjusted and fully adjusted Cox models confirmed the results of the propensity score approach (Table S1).

**Figure 1:**
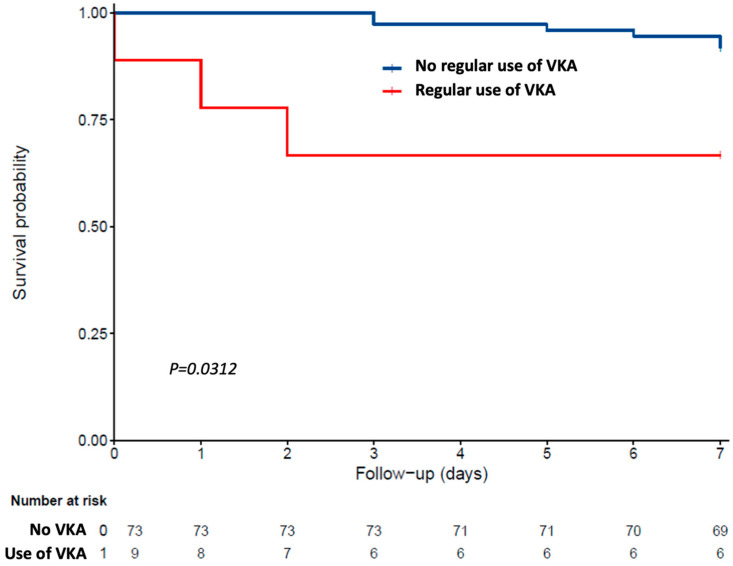
Kaplan–Meier estimates of the cumulative probability of COVID-19 participants’ survival according to the regular use of vitamin K antagonist (VKA) prior to COVID-19 (*n* = 82).

Consistently, Kaplan–Meier distributions, drawn without propensity score adjustment in Figure 1, showed that COVID-19 patients using VKA on a regular basis had shorter survival times than those not using VKA.

## 4. Discussion

The main result of this cohort study is that, irrespective of all measured covariables, the regular use of VKA prior to COVID-19 was associated with a lower survival rate in hospitalized frail elderly patients with COVID-19. This novel finding calls for the careful use of VKA in this population and to prefer anticoagulants that do not disrupt the vitamin K cycle when there is an indication for anticoagulation. This lesson for routine practice is consistent with current guidelines encouraging to use of DOACs first-line after age 75 [11].

To our knowledge, we provide here the first data examining the association of the regular use of VKA prior to COVID-19 with the survival rate of COVID-19 patients. One previous cohort study has examined the association of the use of anticoagulants (i.e., VKAs, DOACs or heparins) prior to (and during the earliest stages of) COVID-19 with the outcomes of COVID-19 [14]. The authors found from 3772 patients (*n* = 241 using anticoagulants, without further clarification on the use of VKA specifically) that the anticoagulation brought no benefit in preventing severe forms of COVID-19 and in reducing all-cause mortality [14]. Moreover, taking into account the ongoing uncertainty regarding the role of a procoagulant state in the pathophysiology of severe COVID-19, some have opted to use anticoagulants as a treatment option for patients with already diagnosed COVID-19. For instance, prophylactic heparin was used in 99 patients with COVID-19 in Wuhan, China, and was associated with improved survival in the specific subgroup with a sepsis-induced coagulopathy score ≥ 4 [15]. However, the rate of prophylactic anticoagulation was rather low for some poorly defined reason [15]. In addition, the use of anticoagulants of all classes was associated in 4389 patients hospitalized for COVID-19 in 5 New York hospitals, with reduced mortality and reduced use of intubation [16]. However, a significant proportion of patients had taken more than one anticoagulant during hospitalization in this study, which precluded a direct comparison between anticoagulants and prevented from determining the selective effect of VKAs. Moreover, the absence of randomization in this observational study precluded the inference that anticoagulation provided a benefit in the observed reduction in mortality and intubation. The reasons why the patients were not treated with anticoagulants may be related to their underlying frailty or to the concomitant presence of severe bleeding history, thrombocytopenia or coagulopathy, all of which may alter patient prognosis. The benefit of anticoagulation in reducing thrombotic complications should, therefore, be weighed against the risks of adverse effects such as major bleeding, but also the reduction of vitamin K concentrations when taking VKA. Thus, our present results provide novel information by reporting that the long-term use of VKA prior to COVID-19 was specifically associated with increased mortality risk in COVID-19 patients, which should be taken into account in clinical decisions and in the choice of anticoagulant.

How vitamin K status is associated with COVID-19 survival is not fully elucidated. Four mechanisms are likely: regulation of (i) anticoagulation, (ii) lung fibrosis, (iii) inflammation, and (iv) host frailty and comorbidities. First, coagulation is a complex balance between the processes of promoting and dissolving clots. The carboxylation and biological function of coagulation factors II, VII, IX and X depend on vitamin K. A decrease in vitamin K leads to more severely compromised carboxylation of extrahepatic proteins than of vitamin K-dependent hepatic proteins [17], which may paradoxically lead to thrombogenicity [18]. Second, vitamin K is involved, via the matrix Gla protein (MGP) [19], in the regulation of calcifications in the arterial walls and in the lung matrix [20,21]. Since MGP needs vitamin K to become biologically active, the under-carboxylation of MGP due to 6 weeks of treatment with VKA was associated with accelerated arterial calcification [22]. It is also suspected that the compromise of the MGP carboxylation by VKA is involved in the onset of idiopathic pulmonary fibrosis [23,24] since elastic fibers have a strong affinity with calcium [25]. Of note, uncarboxylated MGP has been shown to worsen outcomes mainly in atherosclerotic, diabetic and hemodialysis patients, in whom supplementary vitamin K2 treatment may reduce inactive MGP levels [26]. Lung fibrosis may also be exacerbated with VKA by preventing the activation of anticoagulant proteins C and S, which both have antifibrotic properties [27,28]. Third, vitamin K exerts an anti-inflammatory effect mediated by the reduction of PGE2, COX2 and IL-6 [29]. Vitamin K deficiency due to the use of VKA is accompanied by an increase in circulating levels of inflammatory cytokines such as IL-6 and C-reactive protein [29]. Similarly, one recent study reported that vitamin K deficiency in male COVID-19 patients was associated with greater IL-6 levels in the general circulation [30]. This suggests that vitamin K deficiency may be involved in the COVID-19 cytokine storm and related fatal outcomes, including ARDS [1]. Fourth, reduced vitamin K status is associated with comorbidities such as hypertension, diabetes mellitus and cardiovascular disease [1,6,30], which are known to contribute to the severity of COVID-19 [1].

We also noted here that female sex was associated with lower 7-day mortality (Table S1). This result is consistent with previous literature that points out a special vulnerability of men to COVID-19 [1]. Thus, this result validates the consistency of our cohort and of our main result, i.e., the association of the regular use of VKA with poorer survival in COVID-19 patients.

The strengths of the present study include (i) the originality of the research question on an emerging infection for which there is no scientifically validated treatment and in which every effort should be made to improve the prognosis, (ii) the follow-up and the detailed description of the participants’ characteristics allowing the use of a propensity-score-weighted Cox proportional-hazards model to measure adjusted longitudinal associations according to regular use of VKA prior to COVID-19, and (iii) the standardized collection of data from a single research center. Regardless, a number of limitations also existed. First, the study participants were restricted to a limited number of hospitalized frail elderly patients, with a relatively low proportion under VKA, who may be unrepresentative of all older adults. Second, although we were able to control for important characteristics that could modify the association, residual potential confounders may still be present such as the serum phylloquinone concentration (which is commonly low in older adults [30]), the dietary vitamin K intake, or the history of INR prior to and during COVID-19. Third, the observational design of our study is less robust than an interventional study and prevents any causal inference, even if using a propensity score, defined as the probability of treatment assignment conditional on baseline covariables, is helpful for estimating the effects of treatments from observational data in an unbiased way [12].

## 5. Conclusions

We were able to report among hospitalized frail elderly patients with COVID-19 an increased mortality risk at day 7 associated with the regular use of VKA prior to COVID-19. Even if the benefits of anticoagulation are indisputable for indications such as atrial fibrillation [11], further larger prospective observational cohorts and randomized clinical trials, preferentially on a variety of adult populations, are needed to clarify the prognosis of COVID-19 among those using VKA on a regular basis compared to those using DOAC, and whether there is a differential effect on the prognosis according to the time of anticoagulant administration prior to or following the diagnosis of COVID-19. Determining whether vitamin K supplementation has an interest in the prevention and treatment of severe COVID-19 will also be important.

## Figures and Tables

**Table 1:** Characteristics and comparison of COVID-19 participants (*n* = 82) separated into two groups according to 7-day mortality.

	Total Cohort (*n* = 82)	7-Day Mortality		*p*-Value *
		No (*n* = 73)	Yes (*n* = 9)	
**Demographical data**				
Age (years), med (IQR)	88 (85–92)	88 (85–92)	89 (87–92)	0.655
Female sex	39 (47.6)	35 (48.0)	4 (44.4)	1.000
GIR score (/6), med (IQR)	4 (2–4)	4 (2–4)	3 (2–4)	0.387
**History and treatment data**				
History of cancer	29 (35.4)	23 (31.5)	6 (66.7)	0.061
Severe undernutrition ^†^	25 (30.9)	20 (27.8)	5 (55.6)	0.126
History of diabetes mellitus	19 (23.2)	18 (24.7)	1 (11.1)	0.677
History of hypertension	52 (63.4)	46 (63.0)	6 (66.7)	1.000
History of cardiomyopathy	44 (53.7)	39 (53.4)	5 (55.6)	1.000
History of myocardial infarction	16 (19.5)	13 (17.8)	3 (33.3)	0.368
History of congestive heart failure	26 (31.7)	23 (31.5)	3 (33.3)	1.000
History of stroke and/or transient ischemic attack	16 (19.5)	14 (19.2)	2 (22.2)	1.000
CHA2DS2-VASc score, med (IQR)	4 (3–5)	4 (3–5)	5 (3–5)	0.957
HAS-BLED score, med (IQR)	2 (2–3)	2 (2–3)	3 (2–4)	0.111
Regular use of vitamin K antagonist prior to COVID-19	9 (11.0)	6 (8.2)	3 (33.3)	0.056
**Hospitalization data**				
Non-sinus heart rhythm on admission	30 (36.6)	28 (38.4)	2 (22.2)	0.475
Use of antibiotics ^‡^	59 (72.0)	52 (71.2)	7 (77.8)	1.000
Use of pharmacological treatments of respiratory disorders ^||^	9 (11.0)	6 (8.2)	3 (33.3)	0.056
Estimated glomerular filtration rate (mL/min) ^§^, med (IQR)	71.6 (52.8–93.1)	71.8 (53.7–92.7)	42.6 (34.2–101.4)	0.244

Data presented as *n* (%) where applicable; CHA2DS2-VASc score for atrial fibrillation stroke risk; GIR: iso resource groups; HAS-BLED score for major bleeding risk; IQR: interquartile range; * between-group comparisons based on Fisher’s exact test or Mann–Whitney Wilcoxon test, as appropriate; ^†^ serum albumin concentration < 30 g/L; ^‡^ quinolones, beta-lactams, sulfonamides, macrolides, lincosamides, aminoglycosides, among others; ^||^ beta2-adrenergic agonists, inhaled corticosteroids, antihistamines, among others; ^§^ estimated using the modification of diet in renal disease (MDRD) study equation.

## Data Availability

Patient level data are freely available from the last author at Cedric.Annweiler@chu-angers.fr. There is no personal identification risk within this anonymized raw data, which is available after notification and authorization of the competent authorities.

## Supplementary Materials

The following are available online at https://www.mdpi.com/2072-6643/13/1/39/s1, Table S1: Univariate and multiple Cox proportional-hazards model showing the hazard ratio for 7-day mortality (dependent variable) according to the regular use of vitamin K antagonist prior to COVID-19 (independent variable), adjusted for participants’ characteristics ( *n* = 82).

## References

[1] Zhou F., Yu T., Du R., Fan G., Liu Y., Liu Z., Xiang J., Wang Y., Song B., Gu X. (2020). Clinical course and risk factors for mortality of adult inpatients with COVID-19 in Wuhan, China: A retrospective cohort study. Lancet.

[2] Cui S., Chen S., Li X., Liu S., Wang F. (2020). Prevalence of venous thromboembolism in patients with severe novel coronavirus pneu-monia. J. Thromb. Haemost..

[3] Tang N., Li D., Wang X., Sun Z. (2020). Abnormal coagulation parameters are associated with poor prognosis in patients with novel coronavirus pneumonia. J. Thromb. Haemost..

[4] Dofferhoff A.S.M., Piscaer I., Schurgers L.J., Visser M.P.J., Ouweland J.M.W.V.D., A De Jong P., Gosens R., Hackeng T.M., Van Daal H., Lux P. (2020). Reduced vitamin K status as a potentially modifiable risk factor of severe COVID-19. Clin. Infect. Dis..

[5] Luo W.-R., Yu H., Gou J.-Z., Li X.-X., Sun Y., Li J.-X., He J.-X., Liu L. (2020). Histopatological Findings in the Explant Lungs of a Patient With COVID-19 Treated With Bilateral Orthotopic Lung Transplant. Transplantation.

[6] Fraser J.D., A Price P. (1988). Lung, heart, and kidney express high levels of mRNA for the vitamin K-dependent matrix Gla protein. Implications for the possible functions of matrix Gla protein and for the tissue distribution of the gamma-carboxylase. J. Biol. Chem..

[7] Ansell J., Hirsh J., Hylek E., Jacobson A. (2008). Crowther M, Palareti G. American College of Chest Physicians: Pharmacology and Management of the Vitamin K antagonists: American College of Chest Physicians Evidence-Based Clinical Practice Guidelines (8th Edition). Chest.

[8] French National Security Agency of Medicines and Health Products (ANSM) Anticoagulants in France in 2012: Inventory and Monitoring. http://ansm.sante.fr/var/ansm_site/storage/original/application/901e9c291a545dff52c0b41365c0d6e2.pdf.

[9] Gąsecka A., Borovac J.A., Guerreiro R.A., Giustozzi M., Parker W.A., Caldeira D., Chiva-Blanch G. (2020). Thrombotic Complications in Patients with COVID-19: Pathophysiological Mechanisms, Diagnosis, and Treatment. Cardiovasc. Drugs Ther..

[10] Vetel J.M., Leroux R., Ducoudray J.M. (1998). AGGIR. Practical use. Geriatric Autonomy Group Resources Needs. Soins Gérontol..

[11] Camm A.J., Kirchhof P., Lip G.Y., Schotten U., Savelieva I., Ernst S., Van Gelder I.C., Al-Attar N., Hindricks G., Developed with the special contribution of the European Heart Rhythm Association (EHRA) (2010). Guidelines for the management of atrial fibrillation: The Task Force for the Management of Atrial Fibrillation of the European Society of Cardiology (ESC). Eur. Heart J..

[12] Li F., Morgan K.L., Zaslavsky A.M. (2018). Balancing Covariates via Propensity Score Weighting. J. Am. Stat. Assoc..

[13] Flaczyk A., Rosovsky R., Reed C.T., Bankhead-Kendall B.K., Bittner E.A., Chang M.G. (2020). Comparison of published guidelines for management of coagulopathy and thrombosis in critically ill patients with COVID 19: Implications for clinical practice and future investigations. Crit. Care.

[14] Tremblay D., Van Gerwen M., Alsen M., Thibaud S., Kessler A., Venugopal S., Makki I., Qin Q., Dharmapuri S., Jun T. (2020). Impact of anticoagulation prior to COVID-19 infection: A propensity score–matched cohort study. Blood.

[15] Tang N., Bai H., Chen X., Gong J., Li D., Sun Z. (2020). Anticoagulant treatment is associated with decreased mortality in severe coronavirus disease 2019 patients with coagulopathy. J. Thromb. Haemost..

[16] Nadkarni G.N., Lala A., Bagiella E., Chang H.L., Moreno P.R., Pujadas E., Arvind V., Bose S., Charney A.W., Chen M.D. (2020). Anticoagulation, Bleeding, Mortality, and Pathology in Hospitalized Patients With COVID-19. J. Am. Coll. Cardiol..

[17] Booth S.L., Martini L., Peterson J.W., Saltzman E., Dallal G.E., Wood R.J. (2003). Dietary Phylloquinone Depletion and Repletion in Older Women. J. Nutr..

[18] Nigwekar S.U., Thadhani R., Brandenburg V.M. (2018). Calciphylaxis. N. Engl. J. Med..

[19] Chatrou M.L., Winckers K., Hackeng T.M., Reutelingsperger C.P., Schurgers L.J. (2012). Vascular calcification: The price to pay for anti-coagulation therapy with vitamin K-antagonists. Blood Rev..

[20] Price P.A., Buckley J.R., Williamson M.K. (2001). The amino bisphosphonate ibandronate prevents vitamin D toxicity and inhibits vita-min D-induced calcification of arteries, cartilage, lungs and kidneys in rats. J. Nutr..

[21] Rucker R.B. (1974). Calcium Binding to Elastin. Adv. Exp. Med. Biol..

[22] Schurgers L.J., Spronk H.M., Soute B.A., Schiffers P.M., DeMey J.G., Vermeer C. (2007). Regression of warfarin-induced medial elastocal-cinosis by high intake of vitamin K in rats. Blood.

[23] Hardie W.D., Korfhagen T.R., Sartor M.A., Prestridge A., Medvedovic M., Le Cras T.D., Ikegami M., Wesselkamper S.C., Davidson C., Dietsch M. (2007). Genomic profile of matrix and vasculature remodeling in TGF-α in-duced pulmonary fibrosis. Am. J. Respir. Cell. Mol. Biol..

[24] Booth A.J., Hadley R., Cornett A.M., Dreffs A.A., Matthes S.A., Tsui J.L., Weiss K., Horowitz J.C., Fiore V.F., Barker T.H. (2012). Acellular normal and fibrotic human lung matrices as a culture system for in vitro investigation. Am. J. Respir. Crit. Care Med..

[25] Basalyga D.M., Simionescu D.T., Xiong W., Baxter B.T., Starcher B.C., Vyavahare N.R. (2004). Elastin degradation and calcification in an abdominal aorta injury model: Role of matrix metalloproteinases. Circulation.

[26] Westenfeld R., Krueger T., Schlieper G., Cranenburg E.C., Magdeleyns E.J., Heidenreich S. (2012). Effect of vitamin K2 supplemen-tation on functional vitamin K deficiency in hemodialysis patients: A randomized trial. Am. J. Kidney Dis..

[27] Urawa M., Kobayashi T., D’Alessandro-Gabazza C.N., Fujimoto H., Toda M., Roeen Z., A Hinneh J., Yasuma T., Takei Y., Taguchi O. (2016). Protein S is protective in pulmonary fibrosis. J. Thromb. Haemost..

[28] Lin C., Von Der Thüsen J., Isermann B., Weiler H., Van Der Poll T., Borensztajn K., Spek C.A. (2016). High endogenous activated protein C levels attenuates bleomycin-induced pulmonary fibrosis. J. Cell. Mol. Med..

[29] Suleiman L., Negrier C., Boukerche H. (2013). Protein S: A multifunctional anticoagulant vitamin K-dependent protein at the cross-roads of coagulation, inflammation, angiogenesis, and cancer. Crit. Rev. Oncol. Hematol..

[30] Anastasi E., Ialongo C., Labriola R., Ferraguti G., Lucarelli M., Angeloni A. (2020). Vitamin K deficiency and covid-19. Scand. J. Clin. Lab. Investig..

