# Supplementary material for: Regular Use of VKA Prior to COVID-19 Associated with Lower 7-Day Survival in Hospitalized Frail Elderly COVID-19 Patients: The GERIA-COVID Cohort Study
Source: Nutrients. 2020 Dec 24;13(1):39. doi: 10.3390/nu13010039 (PMC7824717; doi:10.3390/nu13010039)
Supplement: Supplementary file 1 [file nutrients-13-00039-s001.pdf]

nutrients

Supplementary Table S1. Univariate and multiple Cox proportional-hazards model showing the hazard ratio for 7-day mortality (dependent variable) according to the regular use of vitamin K antagonist prior to COVID-19 (independent variable), adjusted for participants' characteristics (n=82).

	7-day mortality					
	Unadjusted model		Partially-adjusted model		Fully-adjusted model	
	HR [95% CI]	P-value	HR [95% CI]	P-value	HR [95% CI]	P-value
Regular use of vitamin K antagonist	5.41 [1.35;21.70]	**0.017**	11.23 [2.18;57.79]	**0.004**	9.64 [1.60;58.04]	**0.013**
Non-sinus heart rhythm	0.46 [0.10;2.24]	0.339	0.34 [0.07;1.70]	0.188	0.25 [0.04;1.63]	0.148
Age	1.03 [0.89;1.18]	0.717	0.99 [0.85;1.17]	0.942	0.98 [0.79;1.21]	0.833
Female sex	0.86 [0.23;3.21]	**0.0496**	1.00 [0.25;3.94]	0.988	1.02 [0.21;4.97]	0.978
GIR score	0.83 [0.53;1.31]	0.430	0.69 [0.40;1.16]	0.161	0.78 [0.45;1.34]	0.368
History of cancer	3.90 [0.98;15.61]	0.054	-	-	4.66 [0.94;23.05]	0.060
History of hypertension	1.16 [0.29;4.63]	0.836	-	-	1.84 [0.39;8.82]	0.444
History of cardiomyopathy	1.08 [0.29;4.03]	0.905	-	-	0.82 [0.15;4.54]	0.821
Use of antibiotics†	1.35 [0.28;6.50]	0.708	-	-	3.73 [0.48;29.00]	0.208
Use of pharmacological treatments of respiratory disorders‡	4.26 [1.07;17.03]	0.041	-	-	4.81 [0.75;31.00]	0.098
Estimated glomerular filtration rate ǀǀ	0.99 [0.97;1.02]	0.541	-	-	1.00 [0.97;1.02]	0.749

CI: confidence interval; GIR: Iso Resource Groups; HR: hazard ratio; †: quinolones, beta-lactams, sulfonamides, macrolides, lincosamides, aminoglycosides, among others; ‡: beta2-adrenergic agonists, inhaled corticosteroids, antihistamines, among others; ǀǀ: estimated using the Modification of Diet in Renal Disease (MDRD) study equation.
